# A Large Scale Code Resolution Service Network in the Internet of Things

**DOI:** 10.3390/s121115206

**Published:** 2012-11-07

**Authors:** Haining Yu, Hongli Zhang, Binxing Fang, Xiangzhan Yu

**Affiliations:** 1 Research Center of Computer Network and Information Security Technology, Harbin Institute of Technology, Harbin 150001, China; E-Mails: zhl@pact518.hit.edu.cn (H.Z.); yxz@pact518.hit.edu.cn (X.Y.); 2 Institute of Computing Technology, Chinese Academy of Sciences, Beijing 100190, China; E-Mail: bxfang@pact518.hit.edu.cn

**Keywords:** Internet of Things, product code, code resolution service, SkipNet, explicit query, range query

## Abstract

In the Internet of Things a code resolution service provides a discovery mechanism for a requester to obtain the information resources associated with a particular product code immediately. In large scale application scenarios a code resolution service faces some serious issues involving heterogeneity, big data and data ownership. A code resolution service network is required to address these issues. Firstly, a list of requirements for the network architecture and code resolution services is proposed. Secondly, in order to eliminate code resolution conflicts and code resolution overloads, a code structure is presented to create a uniform namespace for code resolution records. Thirdly, we propose a loosely coupled distributed network consisting of heterogeneous, independent; collaborating code resolution services and a SkipNet based code resolution service named SkipNet-OCRS, which not only inherits DHT's advantages, but also supports administrative control and autonomy. For the external behaviors of SkipNet-OCRS, a novel external behavior mode named QRRA mode is proposed to enhance security and reduce requester complexity. For the internal behaviors of SkipNet-OCRS, an improved query algorithm is proposed to increase query efficiency. It is analyzed that integrating SkipNet-OCRS into our resolution service network can meet our proposed requirements. Finally, simulation experiments verify the excellent performance of SkipNet-OCRS.

## 1. Introduction

Nowadays, the Internet of Things (IoT) has become a hot research topic in both the scientific and industrial fields. The IoT is regarded as the next big possibility and opportunity for the Internet [1], which aims to integrate trillions of heterogeneous interconnected physical objects with the virtual world seamlessly to make a full interoperability of these physical objects and sense the physical world anyplace at anytime [2,3]. Physical objects in the IoT cover a broad range of interconnected entities of interest that users or software agents want to track, monitor or interact with in the physical world, such as a human, animal, car, electronic appliance, a closed or open environment like a building, room and river. Each single physical object can be associated with a standardized product code to identify and locate itself in the IoT. The code can be contained in a RFID tag that is attached to the physical object of interest or stored in an embedded device that is equipped with the physical object of interest. In these application scenarios where physical objects cannot integrate to the IoT directly, the product code is the indispensable key identifier for users to capture and share the real-time information about these physical objects. Specially, product codes accompanied with RFID technology have been widely used in supply chains management and collaboration to increase supply chain visibility in today's complex dynamic trading networks. For instance, EPCglobal presents and operates the EPCglobal Architecture Framework to enhance business flows and computer applications through the use of EPCs.

The information resources carried in a product code are very limited as well as implicit. Most information resources about the physical object associated with the code are available globally and in vast amounts in the IoT. Information resources in the context of the IoT can be websites, web services, pieces of data or events, *etc.* These information resources about the physical object of interest can be indexed by the product code and stored in widely distributed, heterogeneous information services controlled by different organizations. In large scale dynamic application scenarios, end users or software agents need a discovery mechanism to discover and access information resources about their interested physical objects immediately, even if they know nothing except the product codes of their interested physical objects. A code resolution service aims to resolve the given product codes for end users or software agents to derive any information resource related to the given codes respecting their access rights [4]. Code resolution service is an indispensable component in the IoT field and receives increasing attentions in recent years. Actually, the similar service has been studied and successfully used in the internet field for many years, such as Domain Name System (DNS). Compared with the Internet, the IoT has some new features, such as huge volumes of data, timeliness, heterogeneity, dynamic, compatibility and security. New features imply new challenges that a code resolution service in the IoT has to face, which results in that most existing resolution services in the internet are not suitable to the IoT. These new challenges can be summed up as the following three major respects:

1. Heterogeneity issue. There are multiple Product Code Standards (PCSs) in the IoT, and each PCS generally has its own code resolution services. Namespaces of these PCSs are overlapped, resulting in resolution conflicts and resolution overloads. Multiple heterogeneous resolution services are deployed in respective close-loop applications independently to form isolated information islands. It is not possible to persuade providers to abandon their existing resolution services to build a global unified code resolution service.
2. Big data issue. The volumes of code resolution records published by different publishers are very massive. Correspondingly, the amount of concurrent parallel queries initiated by potentially millions of requesters is very high, which results in heavy query load and network traffic. Meanwhile, frequent updates and unanticipated changes of resolution records are very common. More effective approaches for resolution records storage and query are required urgently in the presence of big data.
3. Data ownership issue. Data ownership contains information resources ownership and code resolution records ownership. Accompanied by access control mechanisms, the former is managed locally at the ISs, where the owners determine which requesters can access which information resources. The latter is controlled at the code resolution service where one or more providers decide which resolution records are revealed to which requesters. Until now, most code resolution services ignore the access control management of resolution records to make resolution records expose to any requester irrespective of its access rights. Besides, most code resolution services assume that resolution record publishers have to transfer their resolution records ownership to them. However, it is likely that publishers from many different countries and industries are not willing to submit their resolution records to a code resolution service operated by some authority organization or third-party profit organization, because of political and economical factors such as business benefits or privacy protection.

Unfortunately, existing relevant resolution services cannot address these issues very well, and it is unlikely that a global resolution service operated by a single provider to address these issues in the future can (will) be constructed. We consider that a code resolution service network that consists of independent, collaborating code resolution services is required in the IoT. On this premise, in the context of large scale dynamic applications, our contribution mainly contains the following aspects.

1. We provide a comprehensive list of requirements for code resolution service network architecture and code resolution services. Though most of these requirements are gathered from existing work, they are modified or improved according to characteristics of large scale dynamic applications.
2. We propose a uniform code structure to separate the overlapping namespaces of various PCSs and distinguish the publishers of code resolution records without breaking or reconstructing any existing PCS.
3. According to the proposed requirements, we propose a loosely coupled distributed code resolution service network, which is based on an open and compatible architecture to encourage heterogeneous code resolution services to participate and collaborate with each other harmoniously.
4. According to the proposed requirements, we present a code resolution service named SkipNet-OCRS, whose external behaviors follow a novel external behavior model named QRRA mode and internal behaviors are built on an improved SkipNet overlay network. It not only inherits DHT's advantages, but also supports administrative control and autonomy that offers a foundation to enhance security and privacy. For external behaviors, we abstract two external behavior modes from existing code resolution services: DL mode and QR mode. Our proposed QRRA mode combines the advantages of the two modes. For internal behaviors, we present an improved query algorithm. Furthermore, we define other four types of queries for SkipNet-OCRS to cover all possible queries. Meanwhile, the relationships of the five types of queries are analyzed.

The rest of this paper is organized as follows: in Section 2, we give a brief description of existing popular resolution services. In Section 3, we offer some definitions of the terms used in this paper. In Section 4, we consider basic design requirements, which help to define the focus of our work. In Section 5, we present a uniform code structure. In Section 6, we propose a loosely coupled distributed network of heterogeneous, independent, collaborating code resolution services. In Section 7, we describe SkipNet-OCRS in detail, involving a novel external behavior mode and query algorithms. In Section 8, we further discuss the security and privacy issue in SkpNet-OCRS. In Section 9, we evaluate the performance of SkipNet-OCRS via simulations. In the final section, we draw a conclusion of our work and raise several open issues.

## 2. Related Work

In the IoT, code resolution services usually can be classified as follows: (1) Concentrated service and distributed service according to the way of storing resolution records; (2) Hierarchical service and flat service according to the architecture. As for concentrated service, resolution records are stored in a closed and unique repository so as to remove the complexity. However, resolution records in distributed service are stored in an open repository consisting of multiple autonomous nodes that communicate through a network. In this paper, a hierarchical service refers to a tree-based structure like DNS, and a flat service refers to a P2P network using Distributed Hash Tables (DHTs). Since the amount of codes available globally in the IoT is very huge, let alone the resolution records for these codes, absolute centralized and hierarchical services are more suitable for small scale applications than large scale applications. Therefore, a distributed and DHT-based code resolution service is the possible new direction for large scale dynamic applications due to its scalability, decentralization, openness and fault tolerance.

In the following, we will perform a brief overview of existing code resolution services in both literature and industrial practice. Some of them are developed specifically for product code resolution. Though the others are designed for other names such as domain names, it is easy to reuse them for code resolution. Object Name Service (ONS) [5] and EPC Discovery Service (EPCDS) [6,7] are the most famous hierarchical code resolution service defined and operated by EPCglobal. ONS is a DNS-based service, whose purpose is to resolve an EPC for corresponding information resources. ONS neglects the serial number of an EPC, in other words, the granularity of ONS resolution is currently limited to product type, rather than serial-level lookup. EPCDS can be regarded as a search engine for EPC-related information resources, which can provide more abundant and fine granularity resolution for an EPC. Currently, the EPCDS standard is still in development. The BRIDGE project [8] supported by the EU and coordinated by GS1, addresses a wide spectrum of problems related to the implementation of RFID in Europe. BRIDGE describes and analyses eight approaches that could be used for implementing a code resolution service in high-level. Four architectures are proposed and evaluated as promising candidates for large scale resolution services, which are Directory-of-Resources, Notification-of-Resources, Notification-of-Clients and Query Propagation. A code resolution service in BRIDGE can be deployed either as a single server or as a network of federated servers that provide serial-level resolution for EPCs. DDNS [9] and Overlook [10] are both domain name systems which are based on Chord and Pastry DHT network respectively. A basic architecture called OIDA (Object Information Distribution Architecture) using DHT for ONS is proposed in [11]. CoDoNS [12] is implemented based on Pastry and achieves high performance using the Beehive replication framework, which reduces the lookup latency from *O*(log*N*) to *O*(1). These DHT-based resolution services inherit DHT's fault tolerance and load balancing properties, at the same time, eliminate many maintenance problems that cannot be solved in current hierarchical services. However, neglecting the inherent hierarchical structure of codes, the flat structure of DHT brings additional overhead by requiring additional mechanisms to support range query. Actually, each of the two architectures provides unique advantages over the other, while each has its own disadvantages. Based on comparative studies [13–15] about the DNS-based and DHT-based resolution services, we summarize and compare their features in order to attempt to shed light on their advantages and disadvantages, as shown in Table 1.

Hybrid services combining advantages of both DNS-based and DHT-based services have been proposed in several recent studies. Works [16,17] have added features from DNS-based services into different sub-parts of DHT-based services. Similar efforts [18,19] have included features from DHT-based services into DNS-based services. A DHT-DNS hybrid naming system is proposed in paper [20], whose upper level nodes are organized into hierarchical architecture and lower level nodes are organized into DHTs connected with corresponding hierarchical nodes respectively. A hierarchical P2P code resolution service is proposed in [21], whose lower level consists of many disjoint P2P networks and upper level is composed of a single P2P network where each node is regarded as a super node and selected from a P2P network at the lower level. A code can be resolved from top to bottom according to its hierarchical structure. These upper lower nodes or super nodes are more important and may become the bottleneck of these services. Generally, these hybrid services outperform DNS-based and DHT-based services due to their comprehensiveness. However, these approaches lead to hybrid services with unique features. It is questionable whether a hybrid design can always provide the best of both architectures. It is not clear how to extend a hybrid design with its special application scenario.

Some works focus on security and privacy issues of code resolution services. In [22] its authors propose a code resolution service architecture that built a novel query relay mode to ensuring information provider confidentiality. But the architecture will increase complexity of requester greatly. OIDA [11] is a DHT based code resolution service that considers access control and requester privacy. OIDA uses shared key and random salt to enhance requester privacy, uses public-key cryptography to enforce access control. It needs a secure channel to support key predistribution. SHARDIS [23] is a FreePastry based code resolution service, which enhances requester privacy by applying Shamir's secret-sharing on the resolution records of interest and changing shares and their identifiers over time without needing any cryptographic key predistribution. The latency caused by secret-sharing on each byte of resolution records limits its application in latency-critical scenarios.

## 3. Term Definitions

In this section, we give some major definitions used in this paper:

**Definition 1**. An *Information Service* (*IS*) is a web service, which provides a repository to store the information resources and offers an update interface to add, modify or delete the information resources, as well as a query interface to query for its stored information resources.
**Definition 2**. A *code resolution record* is the mapping information of a special code and addresses of ISs that possess any information resource related to the code.
**Definition 3**. A *code resolution service* is a web service that enables a requester to resolve its codes for the information resources of interest and offers possible additional levels of relaying, aggregation and abstraction. Alternative terms in literature are ‘discovery service’, ‘lookup service’, ‘directory service’ and ‘naming service’.
**Definition 4**. A *code resolution service network* is a distributed network, which consists of multiple independent, collaborating code resolution services.
**Definition 5**. A *provider* of a code resolution service is an organization or a company that provides processing power and storage space to run and operate the code resolution service.
**Definition 6**. A *publisher* of a code resolution service is an organization or a company that publishes code resolution records to the code resolution service.
**Definition 7**. A *requester* of a code resolution service is an end user or software agent that initiates a query to the code resolution service. If a requester is also a provider of the code resolution service, it is referred as an *internal requester*, otherwise an *external requester*.

## 4. Requirements for Code Resolution Service Network Architecture and Code Resolution Services

To create a design proposal for a large scale code resolution service network, we need to define the requirements for its architecture and resolution services to fulfill. In this section, we present a comprehensive list of requirements labeled from RQ1 to RQ23, which are highly relevant to the design of a large scale code resolution service network and the wide adoption of a large scale code resolution service. These requirements have been gathered and strengthened based on several works according to large scale application scenarios. They will be used in Section 6 and 7 to reason on the design of our code resolution service network and SkipNet-OCRS. The requirements for the architecture of a large scale code resolution service network are enumerated as follows:

**RQ1: Multiple PCSs coexistence** [4,23,24]. The architecture should allow multiple PCSs coexistence and enable to resolve multiple PCSs without any code resolution conflict. It is advocated to supportthe integration and transformation between different PCSs.
**RQ2: Bootstrapping Strategy**. The architecture should provide a bootstrapping strategy for a requester to discover the correct code resolution services, only by having a code at hand. These code resolution services enable the requester to retrieve its interested information resources. The architecture should serve distinctive concerns of individual requesters.
**RQ3: Openness and Compatibility**. The architecture should be open for code resolution services to join or depart and be backward compatibility with existing legacy code resolution services. Besides, collaboration of independent code resolution services in the architecture should be supported.

The requirements for a large scale code resolution service in our service network are identified as follows:

**RQ4: Ad hoc query** [7]. A code resolution service should support a requester to execute an ad hoc query, as the requester's need arise without predefined and planned communication.
**RQ5: Standing query** [7]. A code resolution service should also support a requester to execute a standing query that may be time-controlled or trigger-controlled for codes of ongoing interest.
**RQ6: Administrative control and autonomy of IS data** [22]. An IS should be in complete control over its IS data that include IS addresses, information resources as well as fine-grained access rights (**RQ6a**) and be able to track the usages upon its IS data (**RQ6b**).
**RQ7: Business relationship independent design** [22]. Changes in business relationships may cause changes of access rights for information resources. These changes should not affect the way in which a requester interacts with a code resolution service. Each IS wants to minimize the access control maintenance effect for information resources sharing.
**RQ8: Complexity of requester**. The complexity of requester should be as low as possible without losing any functionality. A code resolution service should be invoked by not only powerful devices but also resource-constrained devices.
**RQ9: Quality of resolution** [22]. The resolution result for a requester should be complete, correct, and within an acceptable time frame, respecting the requester's access rights defined separately by each IS.
**RQ10: Control of query**. A requester should be able to completely control its queries throughout the code resolution process, especially, after queries have been sent. Generally, these controls should include canceling or retrying a query, denying a query to be forwarded to a list of malicious ISs.
**RQ11: Confidentiality** [22]. Confidentiality can be further divided into two levels: confidentiality of IS data (**RQ11a**) and confidentiality of resolution record (**RQ11b**). The former represents the feature to disclose IS data only to authorized requesters. Similarly, the later represents the feature to disclose resolution records only to authorized requesters.
**RQ12: Authenticity and integrity** [23]. A code resolution service should offer a way to assure data authenticity and integrity. More precisely, a requester's queries, a publisher's resolution records and an IS's returned information resources have to prove their sources and be protected against alteration and forgery during transmission in an unauthorized manner.
**RQ13: Multiple publishers for a code** [4,23]. A code resolution service needs to provide a way for multiple, independent, but authorized publishers to publish their respective resolution records for a code. The code resolution service should be able to identify the publisher of a resolution record to avoid resolution overloads.
**RQ14: Explicit query** [4,23]. Given a code by a requester, a code resolution service should return either information resources related to the particular code or a list of addresses of ISs which possess these information resources.
**RQ15: Range query** [4,23]. If a code has a special hierarchical structure that consists of several fields, given a range on some fields by a requester, a code resolution service should return information resources related to the codes in the given range or a list of addresses of relevant ISs which possess these information resources.
**RQ16: Administrative control and autonomy of resolution records** [4,11]. A publisher of a code resolution service should be in complete control over its resolution records. The controls should include adding, modifying, deleting, tracking the usages upon its resolution records as well as setting fine-grained access rights for its resolution records.
**RQ17: Scalability** [11,22]. A code resolution service should be highly scalable to maintain trillions of resolution records, as well as handle huge query load and network traffic produced by parallel queries from potentially millions of requesters.
**RQ18: Failure tolerance** [11]. A code resolution service should be resilient to single point of failure caused by attacks (specially, denial of service attacks) or server accident failures. This depends on some features in response to these failures, such as dynamic load balancing, self-organize and self-healing.
**RQ19: Flexibility** [20]. A code resolution service should be flexible to support software and hardware resources dynamic reallocation in response to resolving demand fluctuation. For instance, queries for the codes that bound to moon cakes are likely to increase sharply during Chinese Mid-autumn Festival.
**RQ20: Encourage participations** [22]. Actual value of a code resolution service depends on the number of participants. Therefore, a code resolution service should lower the threshold to encourage participation. Participants are encouraged to become a provider, publisher or requester of the code resolution service. The threshold in this context can be related to technical, financial and political obstacles, such as administrative overhead and maintenance cost.
**RQ21: Update propagation** [11]. A code resolution service should support fast update propagation. Authorized changes of code resolution records should be propagated fast throughout the code resolution service, to avoid stale data.
**RQ22: Availability and reliability** [22]. A code resolution service should ensure a high overall service availability and reliability. Availability is defined as a service's immediate readiness for usage whereas reliability refers to the continuity of service over a prolonged period of time.
**RQ23: Requester privacy** [11,22,23]. A code resolution service should ensure a requester's query only reveal to the provider who possesses the resolution record of the requester's interest. Others are unable to infer the requester's interests and behaviors from the usage of the code resolution service.

## 5. A Uniform Code Structure

In this section, we present a uniform code structure for the code resolution service network proposed in the next section. Generally, a code follows a special hierarchical structure defined by some PCS, and it is unique in the namespace of this PCS. The code is regarded as an original code that is defined as follows:

**Definition 1:***Original Code* (*OC*) is a code that belongs to a special PCS advocated by some authority organization. And it is unique in the namespace of the special PCS. However, many organizations advocate their own PCSs, such as EPC standards advocated by EPCglobal, uCode standards advocated by uID center. Coexistence of multiple PCSs will last for a long time in the future. The overlap of namespaces defined by different PCSs may cause code resolution conflicts. It is impossible to persuade these organizations to give up or reconstitute their PCSs, instead support a global uniform PCS defined by third-party legal authorities. In order to separate these overlapping namespaces of different PCSs, we give the definition of standard code as follows.
**Definition 2:***Standard Code* (*SC*) is the global unique identifier of a PCS, which is assigned by a legal authority. The legal authority is responsible for the coordination of SC codes of global PCSs and, in particular, ensuring their stable and secure operation. Multiple, independent, but authorized publishers can publish respective resolution records for a special OC code. It means there may be multiple resolution records come from different publishers for the same OC code in a code resolution service network. These resolution records may be distributed over several different resolution services or different nodes in a resolution service. It is infeasible to rely solely on the OC code to recognize the publisher of a resolution record. Coexistence of multiple publishers for an OC code may generate overloaded resolution results, since the small number of resolution records of interest may be drowned in vast irrelevant resolution records. In order to identify the publisher of a resolution record to avoid the resolution overloads, we introduce the definition of company code as follows.
**Definition 3:***Company Code* (*CC*) is the global unique identifier of a company which is a potential publisher in a code resolution service. It is advocated that the CC code of a publisher derives from its domain name after suitably reversing the components of the domain name. As an example, a publisher whose domain name is “*hit.edu.cn*” can be identified by “*cn.edu.hit*”.

Based on the three definitions, in this paper we consider that a code given by a requester is the combination of a SC code, a CC code and an OC code, which is denoted as *sc:cc:oc*, where *sc* depends on the special PCS that *oc* belongs to, *cc* is completely decided by the requester to indicate its intended publisher, *oc* represents the binary representation that bound to the physical object of the requester's interest. Obviously, the uniform code structure only extends the existing PCSs with SC and CC to separate overlapping namespaces and distinguish publishers without breaking or reconstructing any PCS.

## 6. Architecture of a Large Scale Code Resolution Service Network

The large scale code resolution service network presented in this paper is a loosely coupled distributed network consisting of heterogeneous, independent, collaborating code resolution services. Each code resolution service in the network can be operated by one or more providers such as legal authorities, globally operating communities, partner companies themselves, or third-party for-profit organizations. The special architecture of the service network is determined by the following four factors involving technical, political, legal and economical constraints on large scale dynamic code resolution applications.

1. Massive resolution records come from vast publishers and large amounts of parallel queries initiated by potentially millions of requesters require to run a number of large scale resolution services to share the work load and network traffic.
2. There are multiple PCSs advocated by different authority organizations in the IoT. And it is likely that more PCSs will be presented in the future. Major authority organizations propose respective code resolution services that only serve their own PCSs. It is infeasible to persuade them to give up their resolution services to build a single global resolution service that can handle all possible current and future PCSs.
3. Even for the same PCS, it is likely that the publishers from many different countries and industries are not willing to agree on submitting their resolution records to a single global resolution service operated by some authority organization or third-party profit organization, due to a number of political and legal reasons, such as business benefit and privacy protection.
4. On the other hand, the operation of a global resolution service would require powerful computing and storage capacity. The organization that operates the global resolution service would have to be financed by its users to support its operation. It is likely that no one be willing or able to pay for this code resolution service.

**Figure 1.**
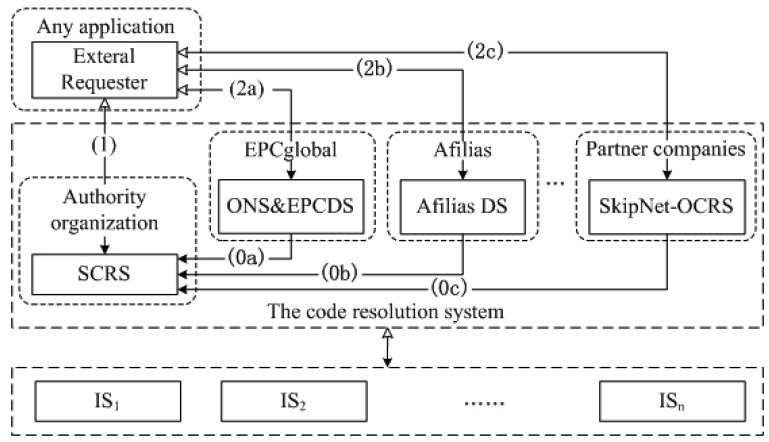
The architecture of the code resolution service network.

Our code resolution service network includes four roles: requester, Standard Code Resolution service (SCRS), Original Code Resolution Service (OCRS) and IS, as illustrated in Figure 1. The code resolution service network is a distributed network consisting of a globally unique SCRS and multiple OCRSs, such as ONS and EPCDS operated by EPCglobal, Affilia DS operated by Afilias and SkipNet-OCRS operated by partner companies themselves.

A requester can initiate a query for one or more interested codes to the code resolution network to discover information resources associated with the codes. The codes will be resolved field by field through the interactions among the requester, the code resolution service network and corresponding ISs.

The SCRS is a globally unique code resolution service operated by some authority organization. The SCRS provides a bootstrapping process for an external requester to locate its intended OCRSs dynamically, only using its SC code (certainly, additional information can also be submitted to achieve more accurate location). In order to be discoverable, OCRSs in the network publish and register their service profiles to the SCRS. A profile has to contain the SC codes of PCSs that the OCRS serves, and may include some additional information such as access endpoints and non-functional descriptions. Generally, the number of PCSs and corresponding large scale code resolution services are not very huge, and the update of their mapping information is not frequent. Therefore, a hierarchical code resolution service like DNS is suitable to the SCRS. In particular, the SCRS can be organized as a domain of DNS, e.g., “.*scrs*”, to inherit DNS's hierarchy. Ignoring the detailed techniques for the connection of each resolution service, the query procedure of SCRS is shown in Figure 1. In step (0), once one or more providers have construct an OCRS for a special PCS, corresponding profile of the OCRS is published and registered to the SCRS. In step (1), an external requester initiates a query that contains a particular SC code to the SCRS. The SCRS provides a list of addresses of OCRSs that can further resolve the requester's CC code and OC code. Then, in step (2), the requester sends queries to these OCRSs in the list to discover the information resources associated with their CC code and OC code. The SCRS can identify and distinguish different PCSs through their assigned SC codes (RQ1). It leverages a dynamical indirect addressing approach to provide a bootstrapping process to enable external requesters to discover and locate corresponding OCRSs (RQ2). The service network architecture ensures openness and compatibility (RQ3). If a requester has been bound to some OCRSs in advance, it can contact with its bonding OCRSs directly without SCRS.

An OCRS is also a kind of code resolution service which is responsible for further resolving a requester's CC code and OC code. There may be various code resolution services in the service network. In the next section, we will propose a large scale OCRS named SkipNet-OCRS.

## 7. A Code Resolution Service Based on SkipNet

To satisfy the requirements for code resolution services in large scale dynamic application scenarios (RQ4~RQ23), we propose a novel OCRS based on SkipNet [25] at a conceptual level, which is named SkipNet-OCRS. In this section, we first give a brief overview of original SkipNet to make this paper self-contained. Then, we describe the external behaviors and internal behaviors of SkipNet-OCRS in detail, respectively.

### 7.1. A Brief Overview of SkipNet

Scalable overlay networks are a useful technique for organizing nodes in a distributed system. For most existing overlays, their primary purpose is to form a DHT network, which allows data to be uniformly diffused over all the participants in the P2P system. Typical examples contain Chord, Pastry, and Tapestry. However, those DHT networks realize perfect load balancing at the cost of controlling data locality. Providing guarantees over data placement and administrative autonomy in overlay networks has been shown to yield improved efficiency, security, reliability and availability [26]. SkipNet is a DHT network based on SkipLists [27], which supports administrative control and autonomy.

SkipNet employs two separate namespaces to identify its nodes and data, which are a string NameID space and a binary number NumericID space. Each node's random choice of ring memberships can be encoded as a unique binary number, which we refer to as the node's NumericID. SkipNet arranges each level of probabilistic SkipLists to form a bidirectional ring where nodes are sorted by their NameIDs. All nodes are connected by the root ring that is the ring at the bottom level. There are one or more rings at higher level, each ring at level *h* is split into *k* disjoint rings at level *h* + 1 by having each node randomly and uniformly choose which of the *k* rings it belongs to. With the splits of rings, the RingIDs are generated to identify rings according to ring memberships, each of which has *h* bits at level *h*. The split of rings is over until there is only one node in each ring and these rings have reached the highest level. After the split terminates, the RingID of a ring at the highest level is the node's NumericID in the ring. Each node in SkipNet needs to construct and maintains several tables to route messages effectively and reliably. Table 2 demonstrates these tables briefly, involving their name, item number, function, where *N* is the number of nodes and *L* is the size of the *Leafset* in SkipNet.

SkipNet supports two message routing algorithms: NameID routing and NumericID routing. The principle of NameID routing is to follow pointers that route closest to the intended destination NameID. Concretely, at each node, a message will be routed along the highest-level pointer in its *R-Table* that does not point past the destination NameID. The message is routed in non-decreasing prefix order and terminates when it arrives at a node whose NameID is prefixed with the destination NameID. The principle of NumericID routing is to follow rings that route closest to the intended destination NumericID. Concretely, the routing operation begins by examining nodes in the root ring until a node is found whose NumericID matches the destination NumericID in the first digit. Then, the routing operation jumps up to this node's ring at level 1 and examines nodes in this ring until a node is found whose NumericID matches the destination NumericID in the second digit. This process repeats until we cannot make any more process, *i.e.*, we have reached a ring at some level *h* such that none of the nodes in that ring share *h*+1 digits with the destination NumericID. The node whose NumericID is numerically closest to the destination NumericID in the ring at this level is the destination of the NumericID routing. The number of message hops for NameID routing and NumericID routing are (log*N*) with high probability.

### 7.2. The External Behaviors of SkipNet-OCRS

The external behaviors describe how the code resolution functionality of SkipNet-OCRS can be achieved in terms of the interactions with SkipNet-OCRS and the functionalities required from relevant requesters or ISs. They represent message exchange schemas between requesters, SkipNet-OCRS and ISs through well-defined interfaces. The external behaviors can be observable from the outside of SkipNet-OCRS. Specifying various roles and interfaces in SkipNet-OCRS is the foundation to understand its external behaviors. Thus, we first describe these roles and interface and then propose three different external behavior modes that SkipNet-OCRS can follow.

#### 7.2.1. Roles and Interfaces in SkipNet-OCRS

SkipNet-OCRS is managed and operated by multiple providers. A provider can also be a publisher or an internal requester. In this paper, we assume that only a provider is able to publish code resolution records to SkipNet-OCRS. Any requester outside SkipNet-OCRS can be regarded as an external requester. A provider of SkipNet-OCRS has to offer a repository to store its own code resolution records. Meanwhile, it should provide several types of interfaces, as listed below:

- At least one *exclusive update interface* for the provider to publish, modify or delete its resolution records manually or for authorized ISs to update resolution records automatically through the notifications that a new OC code is caught for the first time.
- At least one *exclusive query interface* that is only opened to the provider to execute its queries.

In order to allow external requesters to execute their queries, at least one *shared query interface* should be offered for all possible external requesters. Besides these interfaces described above, SkipNet-OCRS may require other interfaces in terms of its external behavior mode (the details will discussed in Section 7.2.2). In SkipNet-OCRS, Ad hoc queries can be handled by these query interfaces directly (RQ4) and standing queries are as well supported through maintaining requesters' registrations and subscriptions for the resolution records related to the codes of ongoing interest in corresponding query interfaces (RQ5).

#### 7.2.2. External Behavior Modes of SkipNet-OCRS

We abstract two external behavior modes from existing code resolution services at a high level, which are Directory Look-up (DL) mode and Query Relay (QR) mode. Combining the advantages of the two modes, we propose a novel external behavior mode named Query Relay & Result Aggregate (QRRA) mode. In rest of this section, we will describe the three modes in details and make a comparison among them through the satisfaction of our proposed requirements to illustrate the advantages of QRRA mode.

##### Mode 1: DL Mode

**Figure 2.**
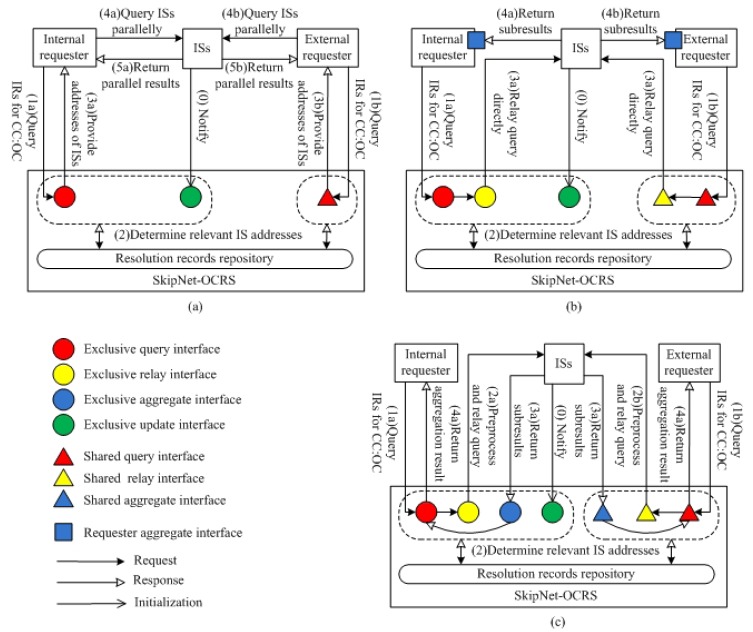
External behavior modes. (**a**) Directory Look-up mode. (**b**) Query Relay mode. (**c**) Query Relay & Result Aggregate mode.

DL mode that is a synchronous request/response paradigm represents the most basic way to provide code resolution functionality. Figure 2(a) illustrates the processes of resolving CC code and OC code for both internal requesters and external requesters in DL mode. (0) Authorized ISs can notify SkipNet-OCRS to update hosted resolution records through corresponding exclusive update interfaces at any time. (1) An internal requester (or external requester) asks for information resources related to a specific CC code and OC code through an exclusive query interface (or a shared query interface). (2) SkipNet-OCRS looks up resolution records to determine addresses of ISs which possess information resources related to the given CC code and OC code. (3) SkipNet-OCRS replies with a list of addresses of relevant ISs to the requester. (4) The requester further queries these ISs whose addresses are in the list directly. (5) These ISs check the requester's access rights, the ISs that have authorized the requester return respective results independently, and other ISs ignore the query immediately without any response. In this mode, the most serious drawback is that ISs are confronted with disclosing privacy about their addresses to unauthorized requesters, since their addresses are sent to any requester irrespective of its access rights. To prevent unauthorized requesters from snooping addresses of ISs, SkipNet-OCRS has to copy access control policies from ISs to implement an additional access layer (meet RQ6a rather than RQ6b). There are two layers of access control about IS data in this mode: one in the ISs and another one in SkipNet-OCRS. However, the two layers approach generates redundancies and increase complexity. Furthermore, it is difficult to maintain the two layers of access control, because the changes of dynamic business relationships between external requesters and owners of ISs directly trigger modifications in the access rights on both two layers. Maintaining permissions on additional access layer is a violation of RQ7. The complexity of requester is very high in the DL mode (a violation of RQ8), because a requester have to invoke relevant ISs, parallelize respective queries to relevant ISs and aggregate incoming ISs' responses completely by itself. Assuming a suitable additional access layer is built on SkipNet-OCRS, a requester can know the exact number of potential ISs. Therefore, complete and correct information resources can be returned parallelly within an acceptable time (RQ9). Additionally, a requester's query is handled confidentially by SkipNet-OCRS without further being passed to ISs, the requester has chance to control its query after receiving the list of addresses of relevant ISs (RQ10).

##### Mode 2: QR Mode

QR mode is essentially an asynchronous request/response paradigm, as depicted in Figure 2(b). In QR mode, a requester needs to implement a callback interface named *aggregate interface*, which is used to receive and aggregate incoming ISs' responses for a single query. SkipNet-OCRS needs to provide at least one *exclusive relay interface* for each provider to propagate its queries to relevant ISs directly, as well as at least one *shared relay interface* for external requesters to forward their queries. The concrete steps in QR mode are described as follows. The step (0), (1) and (2) are almost similar to the steps in DL mode, there is no need to repeat; (3) Once SkipNet-OCRS receives the internal requester's (or external requester's) query, it relays the complete query to relevant ISs directly through an exclusive query interface or (a shared query interface); (4) These ISs check the requester's access rights, and return sub-results directly to the authorized requester through the requester's aggregate interface. The additional access layer is not necessary in QR mode, because SkipNet-OCRS is only responsible to propagate complete queries to respective ISs to avoid resolution records exposed to unauthorized requesters. The responsibility to authenticate requesters is shifted from the SkipNet-OCRS to ISs absolutely. Therefore, ISs are in full control of their own IS data and able to track usages of their IS data (RQ6). Meanwhile, the interactions between a requester and SkipNet-OCRS are not affected by changing business relations (RQ7), because there is no redundant access control maintenance for IS data in SkipNet-OCRS. Unlike DL mode where a requester needs to invoke relevant ISs by itself, SkipNet-OCRS invokes these ISs instead of a requester. But a requester has to be able to receive and aggregate parallel returned sub-results from multiple previously unknown ISs without knowing their exact number. This results in increasing complexity of the requester (a violation of RQ8). Without knowing the exact number of potential ISs, even if a requester waits for a substantial amount of time or tries several times, completeness and correctness of result still cannot be ensured (a violation of RQ9). A requester cannot get any hint to distinguish whether there are some ISs which are relatively slow response or temporarily available, not permanently deniable. Therefore, it is difficult for a requester to set an appropriate timeout to guarantee that most sub-results have been received, ignoring a few replies that still underway. Once a requester initiates a query, it will lose control of the query (a violation of RQ10), because the query has been relayed to relevant ISs immediately.

##### Mode 3: QRRA Mode

QRRA mode combines the advantages of DL mode and QR mode in which SkipNet-OCRS not only preprocesses a requester's query and relays it to relevant ISs, but also receives and aggregates responses from these ISs to respond to the query synchronously. QRRA mode shifts the complexity of query parallelization and ISs' responses aggregation from a requester to SkipNet-OCRS, and creates an additional view of resolution result in a high level for a requester to determine its further operation. Besides these interfaces implemented in QR mode, SkipNet-OCRS needs to provide at least one *exclusive aggregate interface* for each provider to aggregate returned sub-results and create the result view for a single query, as well as at least one *shared aggregate interface* for all external requesters to handle their queries. Figure 2(c) show the code resolution procedure of SkipNet-OCRS in QRRA mode. The steps (0), (1) and (2) are similar to that in DL mode and QR mode. (3) SkipNet-OCRS preprocesses the query to make ISs' responses returned to the corresponding aggregate interface and caches the query temporally. Then, it relays the modified query to relevant ISs through corresponding relay interface. (4) These ISs check the requester's access rights, and return respective sub-results to SkipNet-OCRS through corresponding aggregate interface. A sub-result contains either relevant information resources or access denied notifications. (5) SkipNet-OCRS waits for incoming ISs' responses until its timeout is reached. Then, it aggregates the ISs' responses and returns the aggregated result together with its view to the requester.

SkipNet-OCRS under QRRA mode supports administrative control and autonomy about IS data (RQ6), prevents interactions from being affected by business relationship (RQ7), reduces complexity of requester (RQ8), delivers complete and correct information resources to requesters in low response delay with previously knowing the exact number of available and unavailable ISs (RQ9). To keep response delay as low as possible with a high quality of resolution, SkipNet-OCRS in QRRA mode can set an appropriate timeout to return a possibly incomplete but most aggregated result with an adjunct view that contains a hint that there are some information resources is missing for some reason, such as ISs are relatively slow response, temporarily available or permanently deniable. The view can guide a requester to determine whether retrieve again or ignore the missing information resources. Furthermore, a requester can be in full control of its queries with help of responses aggregation and view creation capabilities offered by SkipNet-OCRS which acts as a mediator for its queries (RQ10).

No matter under which mode, SkipNet-OCRS can be easily expanded to guarantee the confidentiality of information resources by introducing existing authorization and authentication mechanisms (RQ11a), such as Public Key Infrastructures (PKIs) and the authenticity and integrity can be ensured using digital signatures based on certificates of PKIs (RQ12). More details of security and privacy will be discussed in Section 8.

**Figure 3.**
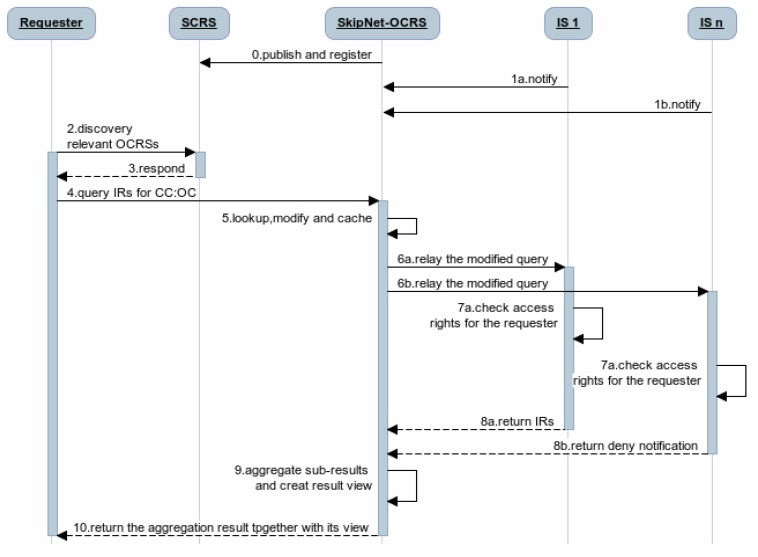
The query procedure of SkipNet-OCRS under QRRA mode.

Obviously, QRRA mode is the most optimal mode for SkipNet-OCRS to follow. Ignoring the detailed techniques for the connection of the each resolution services, the complete query procedure of the code resolution service network that contain SkipNet-OCRS under QRRA mode is depicted in Figure 3. (0) Once one or more providers built SkipNet-OCRS for a special PCS, SkipNet-OCRS should be published and registered to the SCRS. Meanwhile, (1) ISs can notify SkipNet-OCRS to update its resolution records. (2) A requester asks the SCRS to discover relevant OCRSs for its further resolution. (3) The SCRS lookups its repository to find the OCRSs that meet the requester's requirements and returns the endpoints of these OCRSs to the requester. Afterwards, (4) the requester initiates a query to SkipNet-OCRSs to resolve its interested CC code and OC code (here, we only enumerate SkipNet-OCRS in Figure 3 for brevity reasons). (5) SkipNet-SCRS lookups resolution records to find that *IS*_1 and *IS*_*n* are relevant to the query. Meanwhile, it modifies and caches the query. (6) SkipNet-OCRS forwards the modified query to *IS*_1 and *IS*_*n*. (7) The ISs check the access rights of the requester. (8a) Access is granted in *IS*_1, it sends a sub-result that contains information resources related to the CC code and OC code to SkipNet-OCRS in response to the query. (8b) Access is not granted in *IS*_*n*, it returns a sub-result that contains a deny notification to SkipNet-OCRS. (9) SkipNet-OCRS aggregates these sub-results and create a view for them. Finally, (10) the aggregated result together with its view are returned to the requester. Generally, an internal requester of the SkipNet-OCRS executes a query from the step (4), because of the binding relationship between it and SkipNet-OCRS.

### 7.3. The Internal Behaviors of SkipNet-OCRS

The last section only describes the external behaviors of SkipNet-OCRS at a high level, ignoring most of details of its internal behaviors. The internal behaviors describe the structures and workflows inside SkipNet-OCRS, which is the kernel of SkipNet-OCRS and invisible from the outside perspective. We integrate SkipNet into a code resolution landscape to propose a new OCRS named SkipNet-OCRS, which inherits several excellent properties of SkipNet, such as high scalability (RQ17), robustness, fault tolerance (RQ18), flexibility (RQ19), openness (RQ20), maintainability and fast update propagation (RQ21). Especially, it supports administrative control and autonomy of code resolution records. In this section, we will give an elaborate description about internal behaviors of SkipNet-OCRS, involving its topology, resolution record storage, query algorithms, node join and departure algorithm.

#### 7.3.1. The Topology of SkipNet-OCRS

**Figure 4.**
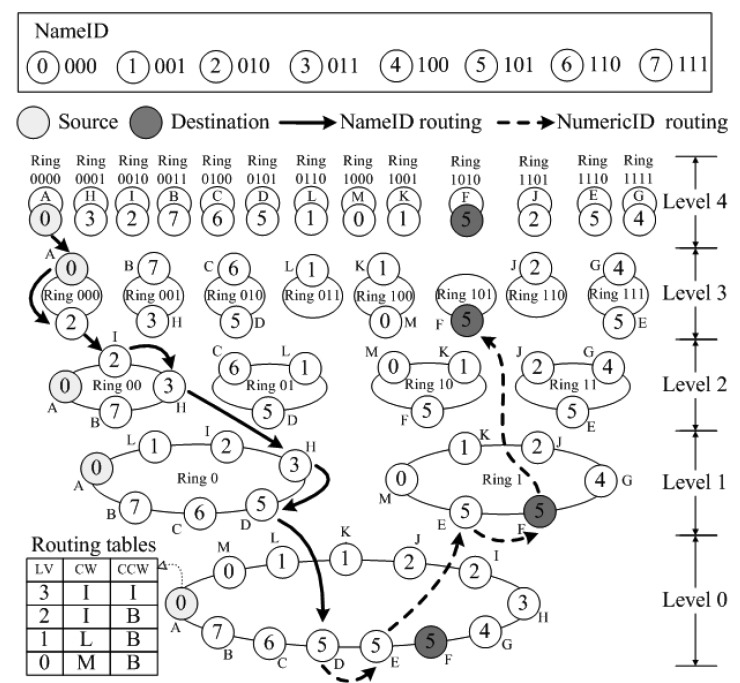
Topology and message routing of SkipNet-OCRS. LV, CW and CCW represent “level”, “clockwise neighbour” and “counterclockwise neighbour” respectively.

The topology of SkipNet-OCRS is similar to that of SkipNet, which is made up by numerous servers (that is regarded as nodes) offered and maintained by respective providers. Each node's NameID consists of two parts: its owner's CC code and its local name. Figure 4 illustrates an example of SkipNet-OCRS with five levels, where NameID is a string derived from a three-digit binary number, NumericID is four-digit binary number and the number of rings generated in each splitting are two. We assume that eight providers offer sixteen nodes totally to compose SkipNet-OCRS. In order to depict more simply, for each node we ignore the local name in its NameID, while only retain the CC code. That is, the nodes belong to a provider have the same NameID, for instance, node *D* and *E* whose NameID is “101” belongs to the provider whose CC code is “101”. In addition, node *D* has “1010” as its NumericID, which belongs to root ring at level 0, ring 1 at level 1,ring 10 at level 2, ring 101 at level 3 and ring 1010 at level 4 respectively.

#### 7.3.2. Resolution Record Storage in SkipNet-OCRS

The nodes in SkipNet-OCRS together provide a repository to store code resolution records. The input code in SkipNet-OCRS is the combination of an OC code and a CC code, which is denoted as *cc*:*oc*. A code resolution record in SkipNet-OCRS can be denoted as a two-tuple <*cc*:*oc*, *IS_addresses*>. For instance, the code resolution record <*cn.edu.hit:010000A8900016F000169DC0*, www.hit.edu.cn/example/IS> indicates that it is associated with “*010000A8900016F000169DC0*” and published by the provider “*cn.edu.hit*”. With regard to resolution record storage, SkipNet-OCRS makes a compromise between load balancing and content locality to implement constrained load balancing (CLB), which is a generalization that combines these two notions: resolution records are uniformly distributed across a well-defined subset of the nodes in SkipNet-OCRS.

Similar to naming of nodes, each resolution record is identified by a NameID and NumericID together. Therefore, an identifier of a resolution record consists of two parts: (1) NameID is equal to the CC code in the resolution record, it specifies the set of nodes over which load balancing should be performed (namely, the CLB domain); (2) NumericID is equal to the hash value of the OC code in the resolution record, it is used to decide which node of the set the resolution record stored in (namely, the CLB suffix). As an example, the identifier of the resolution record given in last example is “*cn.edu.hit:ce8fbdbbc2e662ab5892329232bf471b4e78c768*”. The NameID “*cn.edu.hit*” indicates that load balancing should occur in the CLB domain where each node's NameID starts with prefix “*cn.edu.hit*”. The NumericID “*ce8fbdbbc2e662ab5892329232bf471b4e78c768*” decides that the resolution record is located in the specific node whose NumericID is closest to the resolution record's within the CLB domain. If numerous other resolution records are also stored in the CLB domain “*cn.edu.hit*”, they will be uniformly distributed across all nodes in the CLB domain. Obviously, the publisher of a resolution record can be identified through its NameID (RQ13). Besides, the CC code is an indispensable part in both node's NameID and resolution record's NameID to establish an association between them. This guarantees SkipNet-OCRS to support administrative control and autonomy of resolution records through implementing constrained load balancing (RQ16). More precisely, SkipNet-OCRS has two important properties: (1) *Resolution record locality* refers to the ability for a provider to distribute its published resolution records across its own nodes; (2) *Path locality* refers to the ability to guarantee that message traffic between two nodes within the same CLB domain is routed within that CLB domain only. Resolution record locality can enhance resolution record confidentiality (RQ11b), guarantee that each provider can control its resolution records and nodes completely, track the usages or the queries upon its resolution records. Path locality improves the routing performance due to routing messages within CLB domain generally have low latency, guarantees that a local query is not exposed outside the CLB domain, handles CLB domains disconnections effectively. To improve the robustness of SkipNet-OCRS and avoid the resolution records loss caused by node failures, each resolution record can be stored redundantly in SkipNet-OCRS.

#### 7.3.3. Query Algorithm

**Figure 5.**
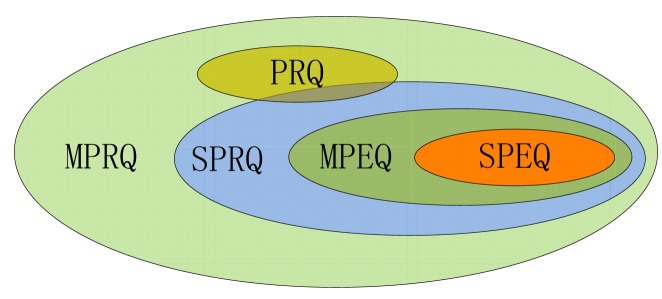
The relationship between SPEQ, MPEQ, PRQ, SPRQ and MPRQ.

Each node in SkipNet-OCRS can act as a source node to initialize a query message for a given *query requirement*. A query requirement consists of two fields: (1) *CC field* refers to an explicit CC code or a specific range of CC code which indicates one or more publishers of interest; (2) *OC field* indicates an explicit OC code or a specific range of OC code. SkipNet-OCRS should support five different types of queries: (1) *Single Provider Explicit Query* (*SPEQ*) for the resolution record associated with an explicit OC code and published by a single provider; (2) *Multiple Provider Explicit Query* (*MPEQ*) for the resolution records associated with an explicit OC code and published by multiple providers whose CC codes are in a given range; (3) *Provider Range Query* (*PRQ*) for all resolution records published by the providers whose CC codes are in a given range; (4) *Single Provider Range Query* (*SPRQ*) for the resolution records associated with the OC codes in a given range and published by a single provider; (5) *Multiple Provider Range Query* (*MPRQ*) for the resolution records associated with the OC codes in a given range and published by multiple providers whose CC codes are in a given range. The containment relationship between these types of queries is depicted in Figure 5. The type of a query can be distinguished by its query requirement. The concrete distinction between the five types of queries is described in Table 3. In this paper, a range of CC code or OC code in a query requirement is represented as a prefix. Give a code *c*=″*e*_1_*e*_2_ ⋯*e_n_*″ (where each *e_i_* corresponds to an element that can be a string or binary number), a code $p=\prime \prime e_{1}^{\prime }e_{2}^{\prime }\cdots e_{m}^{\prime }\prime \prime (m\leq n)$ is called a prefix of *c* if and only if $e_{i}^{\prime }=e_{i}$ for (*i* ≤ *m*). Given a set of codes *S_code_*, a range on *S_code_* identified by a prefix *p* represents the subset of *S_code_*, each code in which is prefixed with *p*.

Generally, exact query (RQ14) contains SPEQ and MPEQ. Range query (RQ15) comprises PRQ, SPRQ and MPRQ. We will discuss the five query algorithms respectively in rest of this section.

##### Query 1: *SPEQ*

In SkipNet-OCRS, the procedure of *SPEQ* for an intended resolution record can be divided into three steps: (1) Construct a query message whose destination NameID is the CC code in the given query requirement and destination NumericID is the hash of the OC code in the given query requirement. (2) Route the message by NameID to find a node in the CLB domain identified by the destination NameID. Then, (3) Route the message by NumericID only among the nodes in the CLB domain to find the node whose NumericID is numerically closest to the destination NumericID, so far, the message routing terminates. The destination node checks the access rights of the requester to determine whether to preprocess the message and relay it to relevant ISs through a corresponding relay interface.

**Figure 6.**
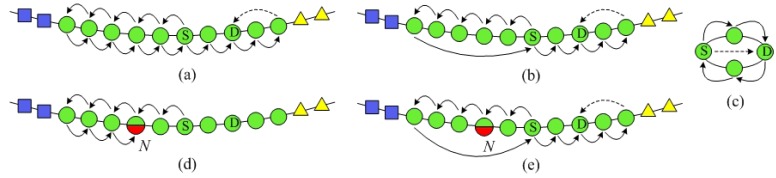
Node structure and message NumericID routing for *SPEQ*. (**a**) Message NumericID routing in a list in original SkipNet. (**b**) Message NumericID routing in a list in SkipNet-OCRS. (**c**) Message NumericID routing in a ring in SkipNet-OCRS. (**d**) Message NumericID routing in a list in original SkipNet with nodes failures. (**e**) Message NumericID routing in a list in SkipNet-OCRS with nodes failures.

*SPEQ* needs to combine SkipNet's NameID routing and NumericID routing algorithm to send a query message to its destination. However, there are two weaknesses in the original work about SkipNet. Firstly, it only proposes NameID routing and NumericID routing algorithm separately. However, how to combine the two routing algorithms to achieve *SPEQ* algorithm is left unstated. Moreover, the node structures where a message may be routed by NumericID aren't identified. Secondly, in original NumericID routing algorithm, there may well be some nodes that have to forward a query message twice, as shown in Figure 6(a). This redundant forwarding problem will inevitably cause additional overload and increase query delay. To solve this problem, we propose an improved NumericID routing algorithm, as shown in Figure 6(b). In the novel algorithm, once a message arrives at a node at a higher ring, the node's information is updated in the query message as the start node of this level. Thus, the bored node in the initial direction can send the message back to the state node directly to avoid redundant forwarding. Intuitively, the improved NumericID routing algorithm can decrease the average hops of original NumericID routing effectively. Furthermore, the improved NumericID routing algorithm can enhance resilience to node failures. As shown in Figure 6(d), in the initial direction, if a node *N* fails after sending a query message to its neighbor, then the message NumericID routing will fail in the list structure, because node *N* can't send the message for second time. The improved NumericID routing algorithm will not be affected by this kind of node failures. The message will be sent to the source node directly, without being sent by the failing node *N* again.

Algorithm 1 presents *SPEQ* algorithm in pseudocode. The query requirement of a SPEQ is *cc'*:*oc'*, which is the argument to the function *SPEQ*. In the function *SPEQ*, the query message is constructed whose destination NameID is assigned with argument *cc'*, destination NumericID is assigned with the hash value of argument *oc'* [see (A) in Algorithm 1] and direction is determined by the lexicographic order between destination NameID and the source node's NameID [see (B) in Algorithm 1]. Then, the function *RouteByNameID* is called to route the message by NameID, which aims to find a node whose NameID is prefixed with destination NameID of the message [see (C) in Algorithm 1]. Along the selected direction, the local node tries to find a candidate of the next node whose NameID is between the local node's NameID and destination NameID from the highest-level pointer in its own R-Table [see (D) in Algorithm 1], and if such a node cannot be found, the level of pointer is decreased [see (E) in Algorithm 1]. If there is no node whose NameID is prefixed with destination NameID, this message routing fails and negative acknowledgment is sent back to the source node [see (F) in Algorithm 1]. Otherwise, assign the direction of the message with clockwise and call the function *RouteByNumericID* to route the message by NumericID in the CLB domain identified by the destination NameID [see (G) in Algorithm 1]. In the function *RouteByNumericID*, the local node examines the number of digits shared between its own NumericID and the destination NumericID to decide the highest level which the message can be routed by NumericID starts from [see (H) in Algorithm 1]. If the length of the common prefix *h* is higher than the level marked in the message, the message jumps to this higher level *h*, at the same time, the local node is regarded as the start node and initial best node whose NumericID is closest to destination NumericID at this level [see (I) in Algorithm 1]. Then, the local node sends the message to its neighbor node along the selected direction at this level *h* [see (J) in Algorithm 1]. Actually, the message tends to be routed in a list structure at most of levels [see Figure 6(b)], but it may be routed in a ring structure at higher levels [see Figure 6(c)]. In a list structure, the message is routed along the list in clockwise direction until arriving at one border node of the CLB domain identified by destination NameID. Then, the border node reverses routing direction to send the message to start node directly at this level [see (K) in Algorithm 1] and continues the message routing in counterclockwise direction until arriving at the other border node [see (L) in Algorithm 1]. In a ring structure, the message is routed only in clockwise direction until arriving at the source node again [see (M) in Algorithm 1]. During the message routing in a list or ring structure described above, if the local node discovers that the NumericID of its neighbor node is closer to the destination NumericID than its own NumericID, the best node saved in the message will be updated to the neighbor node [see (N) in Algorithm 1]. When the list or ring at current level has been successfully traversed and there is no higher level for the message to jump to, the message routing terminates and the best node marked in the message is the destination node, which the intended record resolution is located in [see (O) in Algorithm 1].

**Algorithm 1.** The improved SPEQ.//SPEQ for query requirement *cc*':*oc*'//Initially: msg.ringLvl = -1; msg.startNode = *null*; msg.finalDestination = *false*;SPEQ (*cc*':*oc*', msg) { msg.NameID = *cc*'; msg.NumericID = hash(*oc*'); …(A) if (LongestPrefix(msg.NameID, localNode.NameID) == 0) …(B)  msg.dir = RandomDirection(); else if (msg.NameID < localNode.NameID)  msg.dir = *CCW*;//Counterclockwise else  msg.dir =*CW*;//Clockwise RouteByNameID (msg); …(C)}RouteByNameID (msg) { h = localNode.maxHeight; …(D) while (h >= 0) {  nbr =localNode.RoutingTable[h][msg.dir];  if (LiesBetween(msg.dir, localNode.NameID, nbr.NameID, msg.NameID) ){   if (!CheckIfAlreadyVisited(msg, nbr)) {    msg.AlreadyVisited(localNode);    SendtoNode(nbr, msg); return;   }  }  h--; …(E) } if (!msg.NameID.isPrefixof (localNode.NameID)){ …(F)  NegativeAck(msg); return; } msg.dir = *CW*;//the initial direction of message in NumericID routing is clockwise RouteByNumericID (msg); …(G)}RouteByNumericID (msg) { if (msg.NumericID == localNode.NumericID …(P)  ‖ msg.finalDestination == *true*){ …(O)   ModifyandCacheMessage (msg);   RelayMessage (msg); return; …(R) } if (msg.startNode != null &&localNode == msg.startNode&& msg.dir == *CW*) { …(M)   msg.finalDestination = *true*;   SendtoNode(msg.bestNode);   return; } h = CommonPrefixLen(msg.NumericID, localNode.NumericID); …(H) if (h > msg.ringLvl){ …(I)   msg.ringLvl = h;//The message routing starts from level h   msg.startNode = msg.bestNode = localNode; //Mark the routing start node at level h } if(abs(localNode.NumericID - msg.NumericID)  < abs(msg.bestNode.NumericID – msg.NumericID)){ …(N)   msg.bestNode = localNode; } if(localNode.RoutingTable[h][msg.dir].NameID == msg.NameID){ …(J)   SendtoNode(msg, localNode.RoutingTable[h][msg.dir]); } else if(msg.dir == *CW*){ …(K)   msg.finalDestination = *false*;   msg.dir = *false*;   SendtoNode(msg, msg.startNode); } else if(msg.dir == *CCW*){ …(L)   msg.finalDestination = *true*;   SendtoNode(msg, msg.bestNode); }}

Note that the process described above is on the premise that the node with the destination NumericID in the CLB domain is not found. No matter in which structure and at which level, if the node with the destination NumericID is found, the message routing terminates immediately and the local node is the destination node [see (P) in Algorithm 1]. The destination node modifies and caches the message, then, relays the modified message to relevant ISs according the resolution record associated with *cc'*:*oc'* [see (R) in Algorithm 1].

An instance of SPEQ is illustrated in Figure 4, where the source node *A* (NameID is 000) initiates a query message whose destination NameID is 101 and destination NumericID is 1011. First, the message is routed by NameID (solid lines in Figure 4). Node *A* forwards the message to node *D* (NameID is 101) via node *I* (NameID 010) and node *H* (NameID 011). Then, the message is routed by NumericID (dotted lines in Figure 4). Node *D* (NumericID is 0101) routes the message from the root ring and forwards it to node *E* (NumericID is 1110). The NumericID of node *E* matches the destination NumericID in the first digit, thus, node *E* routes the message in ring 1 at level 1 and forwards the message to node *F* (NumericID is 1010). The NumericID of node *F* matches the destination NumericID in the first three digits to make node *F* route the message in ring 101 at level 3. None of the node in ring 101 belongs to ring 1011, thus, node *F* is selected as the final destination because its NumericID is closest to 1011.

The performance of the improved SPEQ in SkipNet-OCRS is mainly determined by message NameID routing and NumericID routing. Thus, the average number of hops for the improved is *O*(log*N*). Generally, the average number of hops of message NumericID routing is much larger than that of message NameID routing. In this sense, the average number of hops and latency of message NumericID routing is the performance of the improved SPEQ.

##### Query 2: *MPEQ*

A MPEQ can be decomposed to a set of SPEQs, which means that the MPEQ algorithm can be implemented through adding an additional decomposed process to the SPEQ algorithm. Assume that the query requirement of a MPEQ is *cc_pre_*:*oc'* where *cc_pre_* is a prefix that represents the intended range of publishers of resolution records. The steps of a MPEQ are explained as follow: (1) Decompose the range *cc_pre_* in the *CC field* of the query requirement to attain a set of explicit CC codes, which is denoted as *S_pre_* = {*cc*_1_,*cc*_2_,…*cc_L_*}, where *L* is the number of CC codes in the range *cc_pre_*; (2) For *cc_i_* in *S_pre_*, a SPEQ message is constructed and sent to query for the resolution record associated with the *cc_i_*:*oc'*. Obviously, the decomposing of MPEQ is based on the set *S_pre_* which can be attained through our decomposing algorithm. The main idea of this algorithm is to find a node whose NameID is in the given range of CC code as the start node, then traverse the range in the root ring to discover all CC codes.


**Algorithm 2.** Decomposing a range of CC codes.//Initially: msg.S_pre_ = *null*; msg.startNode = *null*; msg.finalDestination = *false*;Decomposing(*cc_pre_*, msg){ msg.prefix= msg.currentNameID = *cc_pre_*; …(A) if (LongestPrefix(msg.currentNameID, localNode.NameID) == 0)  msg.dir = RandomDirection(); else if (msg.currentNameID < localNode.NameID)  msg.dir = *CCW*;//Counterclockwise else  msg.dir =*CW*;//Clockwise RouteByNameID (msg);}RouteByNameID (msg) {  h = localNode.MaxHeight;  while (h >= 0) {  nbr =localNode.RoutingTable[h][msg.dir];  if (LiesBetween(msg.dir, localNode.NameID, nbr.NameID, msg.currentNameID) ){   if (!CheckIfAlreadyVisited(msg, nbr)) {    msg.AlreadyVisited(localNode);    SendtoNode(nbr, msg); return;   }  }  h--; } if (!msg.currentNameID.isPrefixof (localNode.NameID)){ …(B)  SendBack (msg); return; } msg.dir = *CW*;//the initial direction of message traversing is clockwise msg.S_pre_.add(localNode.Name); …(C) msg.currentNameID =localNode.NameID; …(D) msg.startNode = localNode; …(E) TraverseRange(msg);}TraverseRange (msg) { if (msg.finalDestination == *true*){ …(H)  SendBack (msg); return; } if (msg.startNode != null &&localNode == msg.startNode) {  msg.FinalDestination = *true*;  return; } if (msg.prefix.isPrefixof (localNode.RoutingTable[0][msg.dir].NameID)){ …(F)  if (msg.NameID != localNode.RoutingTable[0][msg.dir].NameID){ …(G)   msg.S_pre_.add(localNode.RoutingTable[0][msg.dir].NameID);   msg.currentNameID = localNode.RoutingTable[0][msg.dir].NameID;  }  SendtoNode(msg, localNode.RoutingTable[0][msg.dir]); } else if(msg.dir == *CW*){  msg.finalDestination = *false*;  msg.dir = *CCW*; } else if(msg.dir == *CCW*){  msg.finalDestination = *true*; }}

The decomposing algorithm of MPEQ in pseudocode is shown in Algorithm 2. In the function *Decomposing*, a message whose prefix and current NameID are both initialized as argument *cc_pre_* [see (A) in Algorithm 2]. The selection of initial direction of the message is similar to that in Algorithm 1. The function *RouteByNameID* is invoked to find a node whose NameID is prefixed with the prefix of the message. If there is no such node, the message with an empty set is sent back to its source node to indicate there is no CC code in the range [see (B) in Algorithm 2]. Otherwise, the local node's NameID is added to the set in the message [see (C) in Algorithm 2] and assigned to the current NameID of the message [see (D) in Algorithm 2]. Meanwhile, the local node is regarded as the start node of the message in root ring [see (E) in Algorithm 2]. The function *TraverseRange* is invoked, where the message traversing starts from the start node in the root ring. Along the selected direction, if the neighbor node's NameID is prefixed with the prefix of the message, the message will be forwarded to the neighbor node [see (F) in Algorithm 2]. Furthermore, under this condition, if the neighbor node's NameID is not equal to the current NameID of the message, it means than a new explicit CC code in the range is found. Thus, the neighbor node's NameID is regarded as a new explicit CC code and added to the set in the message. Meanwhile, Current NameID of the message is updated to the new CC code [see (F) in Algorithm 2]. When the range in the root ring has been traversed successfully, the message with the set of explicit CC codes in the range is sent back to its source node [see (H) in Algorithm 2]. The average number of hops for the decomposing algorithm is similar to NameID routing, which is *O*(log*N*). Therefore, the average number of hops for the MPEQ is *O* [(*M* + 1) × log*N*], where *M* is the number of CC codes in a given range.

##### Query 3: *PRQ*

**Figure 7.**
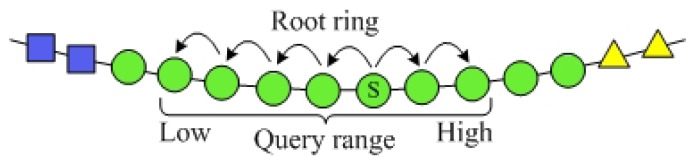
Node structure and message routing for SRQ.

In SkipNet-OCRS, nodes at each ring are sorted by NameID and resolution records whose NameID share common prefixes are stored over contiguous ring segments which these prefixes are closest to or equal to. This node topology and resolution record storage make convenience to support range query on NameIDs of the resolution records, *i.e.*, given a range of CC code that identified by a prefix *cc_pre_*, we can get all resolution records whose publishes are in the range in SkipNet-OCRS. Therefore, the PRQ can be implemented in the SkipNet-OCRS directly. The principle of the PRQ is routing the message to a node whose NameID is in the given range. The message is forwarded from this node along root ring in both two directions at the same time (as illustrated in the Figure 7). Each node in the given range relays the PRQ to relevant ISs and aggregates the ISs' responses. SkipNet-OCRS guarantees *O*(log*N*) performance of PRQ by leveraging the sorted order of NameIDs. The two directions concurrent message forward could reduce latency of PRQ effectively.

##### Query 4: *SPRQ* and Query 5: *MPRQ*

SkipNet-OCRS doesn't support range queries of OC codes that are SPRQ and MPRQ, because each CLB domain offers a completely flat structure of the key space (NumericID namespace) for its resolution records to be randomly partitioned among participating nodes in the CLB domain. It needs additional mechanisms at the cost of increasing time-space complexity to implement range queries of OC codes in SkipNet-OCRS, these additional mechanisms can be LSH (Locality Sensitive Hashing) [28], SFC (Space Filling Curve) [29], PHT (Prefix Hash Tree) [30], *etc.* SkipNet-OCRS enables to be extended with those additional mechanisms to support SPRQ. However, we do not discuss the details because this is out of scope in this paper. The relationship between SPRQ and MPRQ is similar to that between the SPEQ and the MPEQ, a MPEQ can be decomposed into a set of disjoint SPEQs, where the decomposing algorithm is the same as Algorithm 2.

### 7.4. Node Join and Departure Algorithm

If a company wants to join SkipNet-OCRS to become a provider and publish its own resolution records, it first needs to own a unique and widely accepted CC code to identify it nodes and resolution records. The CC code of a company derives from its domain name after suitably reversing the components of the domain name. Then, it has to initialize one or more new nodes identified by NameID and NumericID to store its resolution records. The NameID of a new node is the combination of its owner's CC code and a local name. On the other hand, the hash value of its NameID or IP can be assigned to its NumericID.

A new node's join process consists of three steps: firstly, (1) the new node routes a message to its NumericID to find the top-level ring that corresponds to its NumericID. Then, (2) the new node finds its neighbors by its NameID within the top-level ring only. Starting from one of these neighbors, the node finds its neighbors in the same way at the next lower level. This process is repeated for each level until the node reaches the root ring. Finally, (3) the new node notifies its neighbors in each ring at each level that it should be inserted next to them. Meanwhile, each neighbor shifts these resolutions records to the new node, whose NameID is a prefix of the new node's NameID and NumericID is closer to the new node's NumericID. The number of hops required by a new node to join SkipNet-OCRS is *O*(log*N*) with high probability.

When a node voluntarily departs from SkipNet-OCRS, it should proactively notify all of its neighbors at each level to repair their pointers immediately, and shift its resolution records to appropriate neighbors. When nodes fail abruptly, SkipNet-OCRS handles the issue by three approaches that inherit from original SkipNet: background repair mechanism, fault tolerance mechanism and recovery mechanism from root ring disconnects.

## 8. Further Discussion on Security and Privacy

In this section, we will give some further discussion about security and privacy in SkipNet-OCRS from four aspects: feasible PKI model, code resolution record security and resolution result integrity, requester privacy, access control.

### 8.1. Feasible PKI Model

In order to guarantee authentication, access control, confidentiality, integrity, nonrepudiation in SkipNet-OCRS, we propose a feasible PKI model in conceptual level to create, manage, distribute, use, store, and revoke public key certificates (PKC) for providers, nodes, requesters and ISs. Generally, the CC codes of major providers are hierarchical and they compose a hierarchical namespace. Thus, the PKI model is preferable to being a system composed of many CAs which are organized as a tree-like structure. There is a root CA operated by a management coordinator and corresponding root certificates from which trust may extend, and the root CA can delegate to top provider CAs, which can delegate to other subordinate provider CAs. Each provider CA is responsible to issue PKCs to its own nodes, requesters and ISs independently. With PKCs, nodes, requesters or ISs can authenticate themselves to prevent malicious nodes from joining SkipNet-OCRS, support access control mechanism or avoid unauthorized resolution record storage caused by unauthorized ISs. In order to guarantee secure communication, a keys sharing scheme for a provider and its nodes is offered in SkipNet-OCRS. In this scheme, a provider CA shares its key pairs corresponding to requesters with its nodes. More concretely, given a node, there is a path from a top provider CA to the node in the tree-like PKI model. Each provider CA on the path needs to share its key pairs with the node.

### 8.2. Code Resolution Record Security and Resolution Result Integrity

SkipNet-OCRS has *resolution record locality* property to ensure that resolution records published by a provider are stored in its own nodes. Therefore, the record resolutions don't need to be encrypted storage to guarantee confidentiality (RQ11b). In order to conceal the addresses of ISs from unauthorized requesters while without coping redundant access control policies to SkipNet-OCRS, a destination node doesn't send any resolution record in response to a requester's query in QRRA mode, even if the requester is authorized to access the resolution record. Instead, the node relays the query to corresponding ISs directly. Therefore, a requester has no need to verify authenticity or integrity of its interested resolution records. However, SkipNet-OCRS has to offer a way for a requester to verify authenticity and integrity of aggregation result generated by the node that relays the requester's query before (RQ12). Based on the keys sharing scheme, the node retrieves the private key of the provider that publishes the resolution record of the requester's interest from corresponding provider CA. With the private key, the node encrypts the hash value of the aggregation result and then attaches the digital signature to the aggregation result. The requester may know the provider's public key in advance, or can retrieve and verify the provider's public key by resorting to corresponding provider CA. With the provider's public key, the requester can indirectly verify the authenticity and integrity of the received aggregation result associated with the digital signature.

### 8.3. Requester Privacy

The major privacy adversaries considered in this paper are malicious nodes that belong to other providers in SkipNet-OCRS. Generally, these malicious nodes would like to “play nicely” and offer regular services, but they may eavesdrop on a requester's query message traffic to infer the requester's interested codes that may reflect strategic intents.

SkipNet-OCRS offers three mechanisms to enhance requester privacy (RQ23). The first mechanism is provided by the *path locality* property of SkipNet-OCRS. When an internal requester queries for its published resolution records, the query message is only routed across the internal requester's nodes. Outside nodes cannot eavesdrop on the internal requester's query message traffic, hence, they cannot obtain the internal requester's interested codes. However, this mechanism is limited to the relationship between a requester and its interested provider. That is, its effect weakens gradually with increase of lexicographical distance between the requester's CC code and the provider's CC code. At worst, this mechanism loses effect when the two CC codes disjoint from each other lexicographically. The second mechanism is to use hash value of an OC code instead of a clear-text OC code in a query message. To some extent, this mechanism avoids sending an interested OC code in clear text across nodes that belong to other providers. In most large scale application scenarios, the namespace of all possible OC codes is huge. However, there may be some application scenarios where the namespace is too small to resist dictionary attacks. A malicious node can generate lookup tables for all possible OC codes to make cryptographic hash function useless. If so, the second mechanism becomes invalid. To address this issue, a shared random salt between a requester and its interested provider is preferable to be appended to an OC code [11,23]. However, an additional secure channel is required to distribute the salt. The third mechanism is achieved by encrypting a requester's PKC and digital signature. A requester encrypts its PCK and digital signature using the public key of the provider that publishes the resolution record of its interest. That means, only the destination node that possesses the resolution record of the requester's interest can decrypt the query message and hence recognize its source requester. Other nodes that capture the query message are unable to know which requester initiates the query.

### 8.4. Access Control

In SkipNet-OCRS, access control can be divided into two levels: access control of IS data and access control of resolution record. The former is managed by ISs to determine which requesters can access which IS data. The later is managed by providers to determine which requesters can access which resolution records. The precondition of access control is authentication of requester. Based on the PKI model, both ISs and providers' nodes are able to authenticate possible requesters. In QRRA mode, the access controls of IS data are completely shifted to ISs. ISs can specify and enforce respective access control policies independently. Furthermore, QRRA mode avoids addresses of ISs to be exposed to unauthorized requesters. SkipNet-OCRS is only responsible for access controls of resolution records. With *resolution record locality* property of SkipNet-OCRS, a provider can enforce special access control policies on its own nodes to protect its resolution records against unauthorized requesters. There are many mechanisms that offer not only the enforcement of access control policies but also the management of its evolution [31,32]. Depending on these mechanisms, different extensions to SkipNet-OCRS can be designed. Furthermore, in view of scalability and flexibility, if a delegated authorization protocol is included in SkipNet-OCRS, a requester is able to authorize other requesters to access its authorized resolution records without revealing its PKC.

## 9. Experimental Results and Analysis

In order to evaluate the performance of SkipNet-OCRS, we use a simple packet-level, discrete event simulator that counts the number of packets sent over a physical link and assigns a unit hop count for each link. The source code of SkipNet and its simulation execution environment are provided by the Systems and Networking Research Group at Microsoft Research. Because we don't consider the physical networks which SkipNet-OCRS overlays on, the simulation execution environment and physical network topology are reused directly to build SkipNet-OCRS. Furthermore, the method of modeling provider sizes has little effect on our examination results. Therefore, we just employ the Zipf-like model of original SkipNet. The hardware of our platform is: Intel E4500 2.20 GHz Dual-core CPU, DDR2 667 4 G Memory. The software development environment is: Visual Studio 2008 in Windows XP SP3. All our experiments were run on a Mercator topology [36], which assigns each node to a single Autonomous System and assigns a unit hop count for each link. Assume that the number of nodes in SkipNet-OCRS is *N*, the number of distinct providers that publish resolution records is *M*, The node size of each provider is chosen according to a *Zipf-like* distribution governed by *x*^−1.25^ + 0.5 and normalized to the number of nodes *N*. Nodes that belong to a provider are located within the same AS of the simulated network topology. Concretely, a “root” physical node is randomly placed within the AS and other candidate nodes are sorted by the distance modeled by a Zipf-like distribution from the root node within the AS.

We measured the performance characteristics of queries using the following evaluation criteria:

**Query Hit Rate** (**QHR**) is the rate between successful queries and overall queries under a special time threshold.
**Average Query Time** (**AQT**) is the average elapsed time required to finish a query.
**Average Query Hops** (**AQH**) is the number of average hops required to route a query message from the source node to the destination via the overlay.

Since the Mercator topology does not provide any link latency, AQT is mainly determined by the hardware which the simulations performed on. In our experiments, the purpose of AQT measure is to determine the timeout threshold that indicates whether a query is successful.

In the following two experiments, we evaluate availability, reliability, load balancing and fault tolerance for SkipNet-OCRS. We ran experiments with 50 providers whose CC codes are stored in a list, *i.e.*, *M* = 50. For each participating node, the format of its NameID is *cc/localname*, where *cc* is selected from the list of providers' CC codes according to their node size and *localname* is a random string. Its NumericID is 128 bits binary string generated by the hash of its NameID. The resolution records in our experiments is randomly generated, the size of which is one hundred times larger than the network scale. For each resolution record, the format of its code is *cc*:*oc*, where *cc* is selected from the list of providers' CC codes randomly and *oc* is a random 96-bit EPC code. The resolution record's NameID is equal to *cc* and its NumericID is the SHA-1 hash of *oc*. We set the timeout threshold as 0.2 second to indicate whether a query is successful. Queries can be divided to two groups: local query and global query. The former means the destination node which the intended resolution record is located in and the source node belong to the same provider. The later is opposite, the destination node and the source node belong to different providers. We randomly selected source nodes to initiate 10 × *N* SPEQs for the same parameters and have computed the average value of measured evaluation criteria. Above simulation method has been executed five times and the average value for five simulations is computed as the final value for the evaluation criteria.

**Figure 8.**
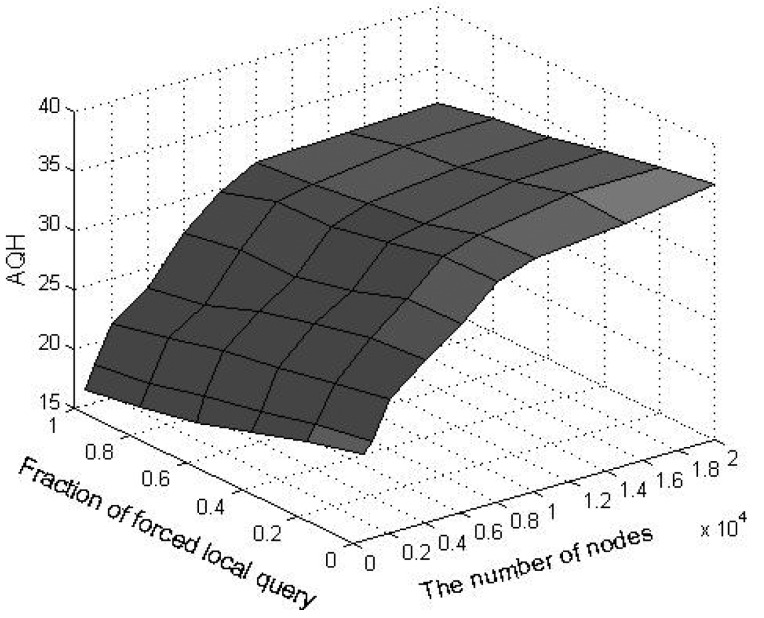
The AQH for SPEQs and network scale under different fraction of forced local queries.

**Figure 9.**
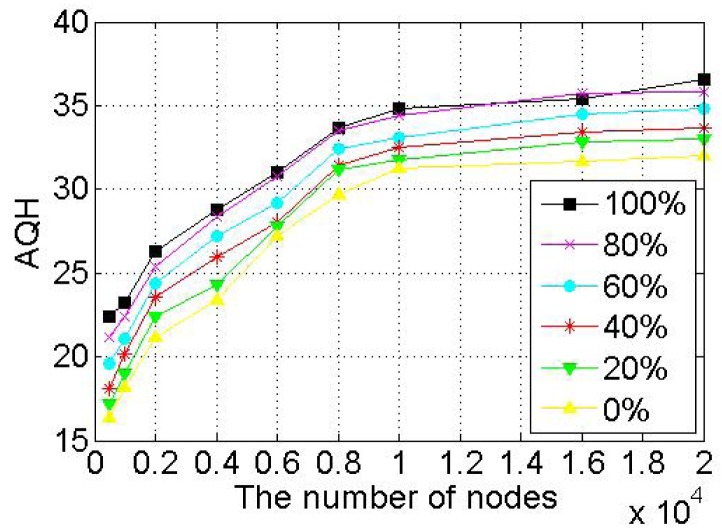
The AQH for SPEQs and the number of nodes.

**Figure 10.**
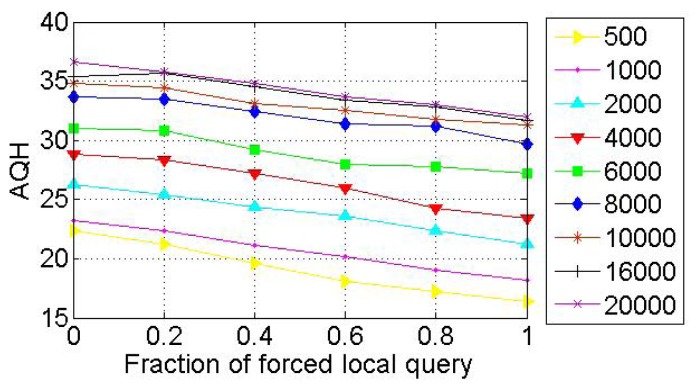
The AQH for SPEQs and fraction of forced local queries.

**Figure 11.**
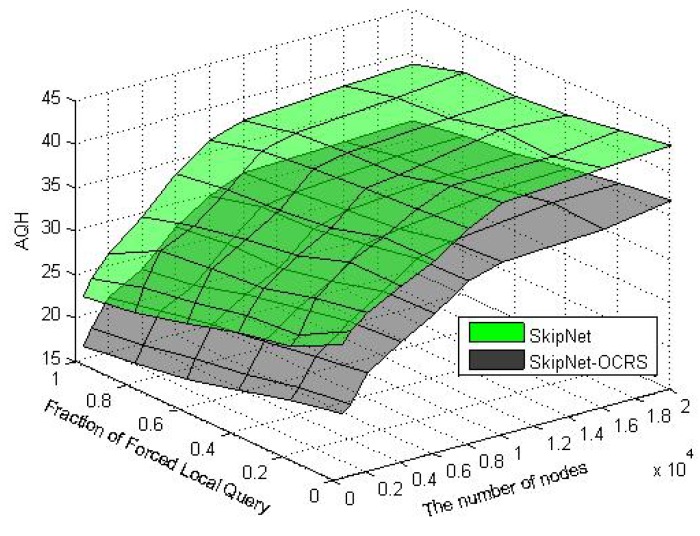
A comparison of the AQH for SPEQ queries between original SkipNet and SkipNet-OCRS.

In the first experiment, we simulated SkipNet-OCRS varying the number of nodes from 500 to 20,000 to measure AQH for SPEQs. Figure 8 presents the relations between the AQH for SPEQs and network scale under different fraction of forced local queries. The *x*-axis indicates network scale, namely, the number of nodes in SkipNet-OCRS, the *y*-axis shows the fraction of forced local queries and the *z*-axis shows the AQH for SPEQs. In order to demonstrate more intuitively and clearly, we illustrate the curves of the relations between the AQH for SPEQs and the number of nodes in Figure 9 and the curves of the relations between the AQH for SPEQs and the fraction of forced local queries in Figure 10. As shown in Figures 8 and 9, we can see that under any the fraction of forced local queries, with the increase of network scale, the AQH increases rapidly when the number of nodes is from 500 to 8,000, and then it grows slowly when the number of nodes is from 8,000 to 16,000, finally it remains almost constant for the rest. For instance, as shown in Figure 9, when the fraction of forced local queries is 60%, the AQH increases less than 13 hops as the number of nodes increases from 500 to 8,000, the AQH increases only about two hops as the number of nodes increases from 8,000 to 16,000, and it would like to increase even more slowly in a larger network scale. It is proved that the AQH is not affected seriously by the network scale, especially, in a large scale scenario where the network scale is very huge. This also means the AQT of SkipNet-OCRS could remain roughly constant in a large scale scenario. If the network latency is low and the network bandwidth is high, the AQT will always be within an acceptable time frame. It verifies that SkipNet-OCRS has excellent capability of scalability, availability and load balancing. Especially, the effectiveness increases significantly as the network scale becomes large. As shown in Figures 8 and 10, under any network scale, the AQH decreases steadily with increase of the fraction of forced local queries. For instance, as shown in Figure 10, when the number of nodes is 20,000, the AQH decreases almost linearly from 36.6 to 32 with the fraction of forced local queries increases from 0 to 1. This indicates the benefits gained by administrative control and autonomy of resolution records. Furthermore, we make a comparison of the AQH for SPEQs between original SkipNet and SkipNet-OCRS, as illustrated in Figure 11. It is obvious that the AQH for our improved SPEQ algorithm of SkipNet-OCRS is smaller than that for SPEQ algorithm of the original SkipNet, regardless of the number of nodes or fraction of forced local queries. This result proves that the improved SPEQ algorithm can decrease the AQH for SPEQs effectively. And the effectiveness of the improved SPEQ algorithm is very stable, not affected by the network scale or the fraction of forced local queries.

**Figure 12.**
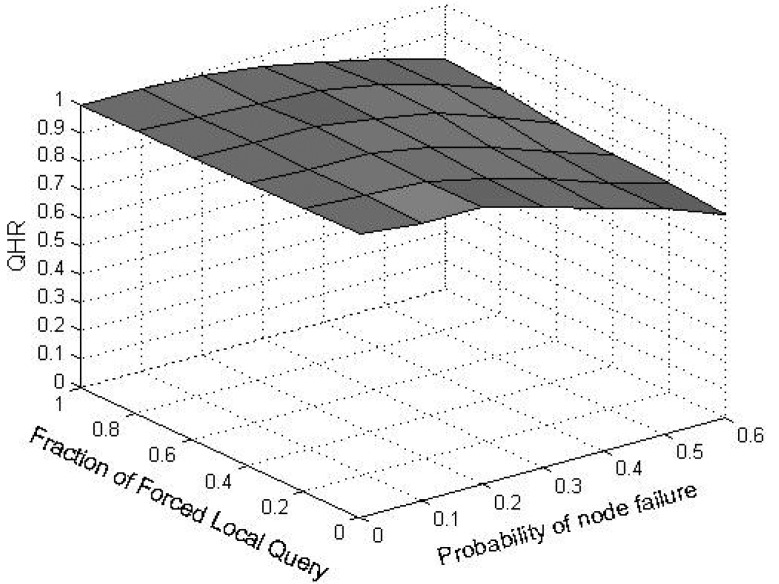
The QHR for SPEQs and probability node failure under different fraction of forced local queries.

**Figure 13.**
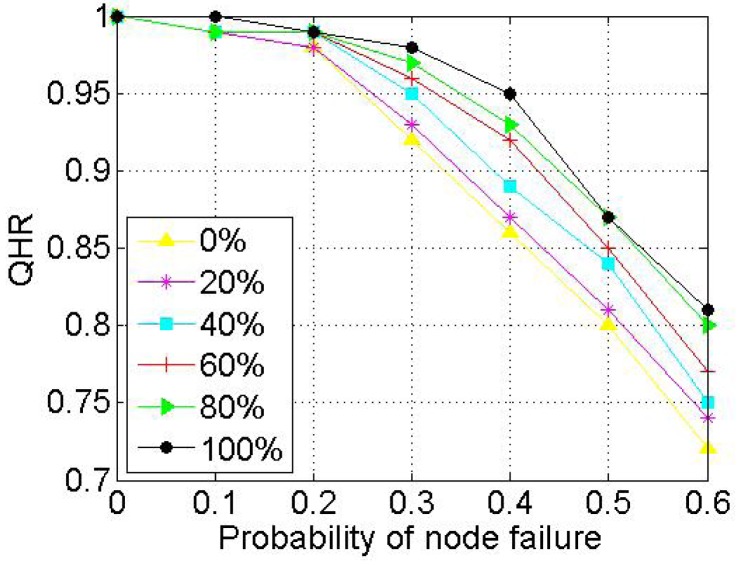
The QHR for SPEQs and probability node failure.

**Figure 14.**
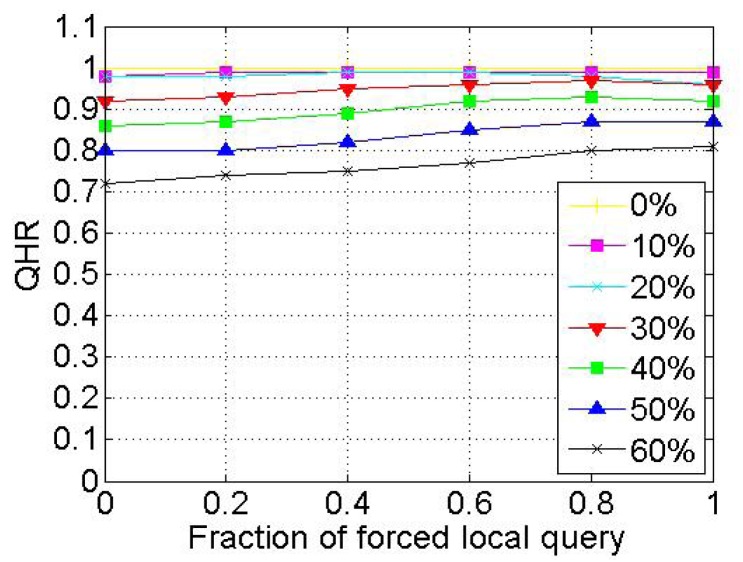
The QHR for SPEQs and fraction of forced local queries.

In the second experiment, we constructed SkipNet-OCRS that contains 20,000 nodes to evaluate its resilience to random node failures. For different fraction of forced local queries, we progressively increased the probability of node failure from 0 to 60% by 10% and measured the QHR for SPEQs. Figure 12 gives the relations between the QHR for SPEQs and probability of node failure under different fraction of forced local queries. The *x*-axis indicates the probability of node failure, the *y*-axis presents the fraction of forced local queries and the *z*-axis shows the QHR for SPEQs. Figure 13 shows the curves of the relations between the QHR for SPEQs and probability of node failure. The experiment results presented in Figures 12 and 13 show that the QHT remains steady and high, when the probability of node failure is less than 20%. Because more and more isolated segments of nodes emerge with node failures, the QHR decreases gradually as the probability of node failure increases when the probability of node failure is more than 20%. However, the QHR doesn't fall much to reach more than 70% even when the probability of node failure comes to 60%. As the results, SkipNet-OCRS is quite resilient to random nodes failure. It verifies that SkipNet-OCRS can provide high availability and fault tolerance. Furthermore, as shown in Figures 12 and 14, the QHR is not affected by node failures when the probability of node failure is less than 20%. Then, the QHR increases slowly as the fraction of forced local queries increases for the rest of the probability of node failure. The rate of this increase accelerates as the probability of node failure increases. This verifies that advantage of administrative control and autonomy of resolution records is increasingly obvious with the probability of node failure increasing, because local queries can be executed successfully in each isolated segment of nodes. This further enhances availability, reliability and fault tolerance in SkipNet-OCRS.

## 10. Summary and Open Issues

Product code is one of the most important identifiers of a physical object in the IoT. It acts as a hinge to integrate a physical object from the physical world into the virtual world. A code resolution service is indispensable to maintain the association between a code and abundant distributed information resources. In this paper, we propose a comprehensive list of requirements and a uniform code structure for code resolution in large scale dynamic application scenarios. We present a loosely coupled distributed code resolution service network consisting of a global unique SCRS and multiple OCRSs. A code following the uniform code structure can be resolved field by field in the code resolution service network. According to the proposed requirements, we propose SkipNet-OCRS which not only inherits DHT's advantages, but also supports administrative control and autonomy of resolution records. We give a comprehensive description about SkipNet-OCRS from its external behaviors and internal behaviors. For external behaviors, three external behavior modes that SkipNet-OCRS can follow are proposed and analyzed. By the comparison of them, QRRA mode combining the advantages of DL mode and QR mode has the most excellent features to fulfill proposed requirements. For internal behaviors, we proposed an improved NumericID routing algorithm to enhance SPEQ efficiency. Beside the improved SPEQ, we define the other four types of queries and their relationship to cover all possible queries. They are MPEQ, PRQ, SPRQ and MPRQ. MPEQ, SPRQ and MPRQ algorithms can derive from the improved SPEQ algorithm. We also further discuss the security and privacy in SkipNet-OCRS. The simulation experiments show that SkipNet-OCRS has good availability, reliability and failure tolerance. SkipNet-OCRS can meet most requirements described in Section 4, as shown in Table 4. The primary limitations of our work are primarily the lack of tests about SkipNet-OCRS in a large scale realistic network environment. The security and privacy in SkipNet-OCRS need to be further considered. Additional protection mechanisms should to be expanded to SkipNet-OCRS to enhance the security and privacy of the implemented SkipNet-OCRS. Furthermore, the communication among independent code resolution services needs to be further investigated, involving communication protocols to support the exchange of information and code transformation between different PCSs. Our future research will focus on these aspects.

## Figures and Tables

**Table 1.** The comparison between DNS-based and DHT-based code resolution services.

	**Hierarchical**	**Flat**	**Exact query**	**Range query**	**Traceability**	**Robustness**	**Resilience**	**Consistency**	**Fast update**	**Flexibility**	**Load balancing**	**Heterogeneity**	**Analyzability**	**Security**	**Openness**	**Legacy friendly**	**Maintainability**	**Average the number of hops**
DNS	●		●	●	●							●	●			●		*O*(1)
DHT		●	●			●	●	●	●	●	●			●	●		●	*O*(log*N*)

**Table 2.** Tables for each node in SkipNet.

**Name**	**Item Number**	**Function**
R-Table	Sparse:*O*(log*_k_N*) Dense:*O*(*k*log*_k_N*)	Key infrastructure for message routing. Store pointers of its neighbor nodes at each level.
Leafset	*L*	Increases fault tolerance and search performance. Store pointers to the *L* additional nodes closest in NameID along the root ring.
P-Table	Sparse:*O*(log*_k_N*) Dense:*O*(*k*log*_k_N*)	Incorporate network proximity, ensure each hop is low in network latency when searching by NameID. Store pointers to the nodes that are roughly the right physical distance (latency) away at each level.
C-Table	*O*(log*_k_N*)	Incorporate network proximity when searching by NumericID in a special domain.

**Table 3.** The query requirements of SPEQ, MPEQ, PRQ, SPRQ and MPRQ.

	**SPEQ**	**MPEQ**	**PRQ**	**SPRQ**	**MPRQ**
**CC field**	A CC code	A range of CC code	A range of CC code	A CC code	A range of CC code
**OC field**	An OC code	An OC code	Null	A range of OC code	A range of OC code

**Table 4.** Fulfillment of requirements. “●” represents a RQ is satisfied completely. “○” represents a RQ is satisfied partly, additional mechanism is needed to full meet the RQ.EBs and IBs represent “external behaviors” and “internal behaviors” respectively.

			**RQ1**	**RQ2**	**RQ3**	**RQ4**	**RQ5**	**RQ6a**	**RQ6b**	**RQ7**	
**SCRS**			●	●	●						
**SkipNet-OCRS**	**EBs**	**DL**				●	●	●			
		**QR**				●	●	●	●	●	
		**QRRA**				●	●	●	●	●	

			**RQ8**	**RQ9**	**RQ10**	**RQ11a**	**RQ11b**	**RQ12**	**RQ13**	**RQ14**	

**SkipNet-OCRS**	**EBs**	**DL**		●	●	○		●			
		**QR**				●		●			
		**QRRA**	●	●	●	●		●			
		**IBs**					●	●	●	●	

			**RQ15**	**RQ16**	**RQ17**	**RQ18**	**RQ19**	**RQ20**	**RQ21**	**RQ22**	**RQ23**

**SkipNet-OCRS**		**IBs**	○	●	●	●	●	●	●	●	●
